# Interplay of Murine Gammaherpesvirus 68 with NF-kappaB Signaling of the Host

**DOI:** 10.3389/fmicb.2016.01202

**Published:** 2016-08-17

**Authors:** Brandon Cieniewicz, Alexis L. Santana, Nana Minkah, Laurie T. Krug

**Affiliations:** Department of Molecular Genetics and Microbiology, Stony Brook University, Stony BrookNY, USA

**Keywords:** NF-kappaB, gammaherpesvirus, pathogenesis, latency

## Abstract

Herpesviruses establish a chronic infection in the host characterized by intervals of lytic replication, quiescent latency, and reactivation from latency. Murine gammaherpesvirus 68 (MHV68) naturally infects small rodents and has genetic and biologic parallels with the human gammaherpesviruses (gHVs), Kaposi’s sarcoma-associated herpesvirus and Epstein–Barr virus. The murine gammaherpesvirus model pathogen system provides a platform to apply cutting-edge approaches to dissect the interplay of gammaherpesvirus and host determinants that enable colonization of the host, and that shape the latent or lytic fate of an infected cell. This knowledge is critical for the development of novel therapeutic interventions against the oncogenic gHVs. The nuclear factor kappa B (NF-κB) signaling pathway is well-known for its role in the promotion of inflammation and many aspects of B cell biology. Here, we review key aspects of the virus lifecycle in the host, with an emphasis on the route that the virus takes to gain access to the B cell latency reservoir. We highlight how the murine gammaherpesvirus requires components of the NF-κB signaling pathway to promote replication, latency establishment, and maintenance of latency. These studies emphasize the complexity of gammaherpesvirus interactions with NF-κB signaling components that direct innate and adaptive immune responses of the host. Importantly, multiple facets of NF-κB signaling have been identified that might be targeted to reduce the burden of gammaherpesvirus-associated diseases.

## Gammaherpesvirus Infection and Associated Malignancies

### Features of Gammaherpesviruses

The gammaherpesviruses (gHVs) represent an ancient and highly successful family of viruses that coevolved with their hosts over the course of 60–80 million years (Davison, 2002). As a subfamily of the *herpesviridae*, the *gammaherpesvirinae* are characterized by an encapsidated double-stranded DNA genome that encodes 70–80 open reading frames (Parker et al., 1990; Russo et al., 1996; Virgin et al., 1997). In addition to protein coding genes, the gHVs encode non-coding RNAs including miRNAs (Pfeffer et al., 2004, 2005). Herpesvirus virions are surrounded by a lipid envelope that contains numerous glycoproteins that mediate entry into the cell. Another characteristic of the herpesvirus virion is the tegument, a structured proteinaceous layer located between the capsid and the lipid envelope. Tegument proteins are delivered into the cytoplasm of the infected cell immediately upon infection and many play crucial roles in early infection.

A hallmark of herpesvirus infection, including that of the gHVs, is the ability to switch between two distinct phases: lytic infection and latency. Lytic infection is characterized by expression of a majority of viral genes in a regulated cascade of gene expression, replication of viral DNA as linear concatemers, and production of infectious virions. Latency is defined by extremely restricted viral gene expression, the maintenance of the viral genome as a circular non-integrated episome tethered to the cellular genome (Yates and Guan, 1991; Ballestas et al., 1999; Lee et al., 1999; Collins et al., 2002; Habison et al., 2012), and the ability to switch from latent infection to productive virus infection, a process known as reactivation. GHVs infect a wide range of cell types, including epithelial cells (Sixbey et al., 1983, 1984), endothelial cells (Boshoff et al., 1995), monocytes (Weck et al., 1999b), and lymphocytes (Alfieri et al., 1991; Sunil-Chandra et al., 1992a) (**Table 1**). The predominant cellular reservoir of latency is lymphocytes; the human gHVs target the mature B cell compartment (Ambroziak et al., 1995; Babcock et al., 1998; Hassman et al., 2011).

**Table 1:** Comparison of select gammaherpesviruses.

	Murine gammaherpesvirus 68 (MHV68)	Herpesvirus saimiri (HVS)	Kaposi’s sarcoma-associated herpesvirus (KSHV)	Epstein–Barr virus (EBV)
Formal name	Murid herpesvirus 4	Saimiriine herpesvirus 2	Human herpesvirus 8	Human herpesvirus 4
Classification	Rhadinovirus	Rhadinovirus	Rhadinovirus	Lymphocryptovirus
Natural host	Wood mice	Squirrel monkey	Humans	Humans
Transmission	Likely sexual	Likely by contact, fomites, aerosol	Saliva, sexual	Saliva, sexual
Associated diseases	Increased incidence lymphoproliferative disease, lymphoproliferative disease and fibrosis in immune deficient mice	Hematologic malignancies in non-native new world primates	Primary effusion lymphomas, Kaposi’s sarcoma, Multicentric Castleman’s disease, AIDS-associated lymphoproliferative disease	Infectious mononucleosis, Burkitt’s lymphoma, gastric carcinoma, nasopharyngeal carcinoma, Hodgkin’s disease, post-transplant and AIDS-associated lymphoproliferative disease
Cell targets	Endothelial cells, epithelial cells, fibroblasts, B lymphocytes, dendritic cells macrophages	Epithelial cells, T lymphocytes	Endothelial cells, B lymphocytes, monocytes, epithelial cells	Epithelial cells, B lymphocytes
Major latency reservoir	B lymphocytes, Macrophage cells	T lymphocytes	B lymphocytes	B lymphocytes
Latency maintenance	Episome tethering to host chromosome via LANA, chromatin silencing	Episome tethering to host chromosome via LANA, chromatin silencing	Episome tethering to host chromosome via LANA, chromatin silencing	Episome tethering to host chromosome via EBNA1, chromatin silencing
Triggers of reactivation	Terminal differentiation to plasma cells, inhibition of histone deacetylases, TLR stimulation, induced expression of viral transactivator RTA	Inhibition of histone deacetylases, induced expression of viral transactivator RTA	Inhibition of histone deacetylases, induced expression of viral transactivator RTA	Terminal differentiation to plasma cells, inhibition of histone deacetylases, induced expression of viral transactivators Zebra and RTA


### The Human Gammaherpesviruses Are Associated with Cancer

Within the gHVs there are two known human gHVs; EBV (Epstein–Barr virus, human herpesvirus-4), is the prototypical member of *lymphocryptoviridae*, and KSHV (Kaposi’s sarcoma-associated herpesvirus, human herpesvirus-8), is the prototypical member of the *rhadinoviridae*. EBV infects over 95% of adult humans, while KSHV infection varies by geographical location and HIV infection status. EBV infection is associated with the development of Burkitt’s lymphoma, Hodgkin’s lymphoma, and nasopharyngeal carcinoma (**Table 1**). KSHV infection is the etiological agent of Kaposi’s sarcoma and is associated with primary effusion lymphoma and multicentric Castleman disease (Verma and Robertson, 2003; Cesarman, 2014). Immunocompromised patients, such as individuals co-infected with HIV and either gHV, have a greatly increased incidence of malignancies (Simard and Engels, 2010; Jha et al., 2016). Despite the effectiveness of HAART therapy in controlling HIV, both gHV-induced lymphomas remain the most common cancer-related cause of death in HIV-infected patients (Simard et al., 2011). EBV infection also causes post-transplant lymphoproliferative disorder (PTLD). Immunosuppressive drugs used to prevent transplant rejection impair T cell control of the latent infection, leading to the emergence of EBV-infected cells with a less restricted latency program that can drive proliferation of virally infected cells (Landgren et al., 2009; Rasul et al., 2012; Tse and Kwong, 2015).

## Murine Gammaherpesvirus 68 (MHV68) Infection of Mice As A Model System of Gammaherpesvirus Pathogenesis

### Value of a Small Animal Pathogen System

Due to the ineffectiveness of current antiviral therapies in combating latent infections and the lack of vaccines, a detailed understanding of virus–host interactions is needed for the design of targeted therapeutics against gHVs. Cell culture-based studies of the human gHVs have defined the role of many viral and host factors during infection. The mechanisms by which EBV and KSHV promote proliferation and survival of the host cell are likely major risk factors for the development of lymphomas. However, studies on the role of virus–host interactions and how they influence pathogenesis in the host have been hampered due to the inability to perform synchronous *de novo* infection in cell culture and the lack of tractable small animal models due to strict host tropism. Thus, a natural gammaherpesvirus pathogen of murid rodents provides a relevant and powerful model system for assaying factors that affect gHV pathogenesis (Simas and Efstathiou, 1998; Blackman et al., 2000; Speck and Ganem, 2010; Barton et al., 2011).

### Murine Gammaherpesvirus 68 Is Endemic to Murid Rodents

Murine gammaherpesvirus 68 (MHV68, formally identified as murid herpesvirus 4) is a natural pathogen of murid rodents used to study virus–host interactions in the context of a whole animal. MHV68 was originally isolated from bank voles in the former Soviet republic of Czechoslovakia (Blaskovic et al., 1980), and has since been identified in yellow-necked wood mice in England (Blasdell et al., 2003), indicating that MHV68 may be endemic to European rodent populations. MHV68 productively infects, and establishes latency in, all tested strains of *Mus musculus*.

### Genomic Similarities to Primate Gammaherpesviruses

Murine gammaherpesvirus 68 shares genomic, biologic, and pathologic properties with primate and human gHVs (**Table 1**). MHV68 is classified as a *Rhadinovirus* along with KSHV and herpesvirus saimiri (HVS, saimiriine herpesvirus 2). The genome of MHV68 is ∼128 kb, and encodes for an estimated 80 ORFs that are largely organized in gene blocks similar to the genomes of HVS, KSHV, and EBV (Efstathiou et al., 1990a,b; Virgin et al., 1997; Simas and Efstathiou, 1998). Transposon mutagenesis screening of MHV68 genes identified a number of genes essential for virus growth that are conserved within the gHV family (Moorman et al., 2004; Song et al., 2005).

As found for other gHVs, MHV68 encodes genes that were likely acquired from the host (Virgin et al., 1997) including a viral homolog of the cellular anti-apoptotic bcl-2 protein (vBcl-2, MHV68 *M11*), a homolog of an IL-8 receptor (vGPCR, MHV68 *ORF74*), cyclin D (v-cyclin, MHV68 *ORF72*), and the complement regulatory protein (MHV68 ORF4; Virgin et al., 1997). MHV68 M11 exhibits both anti-apoptotic and anti-autophagic functions (Ku et al., 2008; Sinha et al., 2008; Xiaofei et al., 2009), and functions to promote the survival of infected immature B cells (Coleman et al., 2014). MHV68 expresses the vGPCR (ORF74), a chemokine receptor with homology to human CXCL2, during latency (Virgin et al., 1997; Wakeling et al., 2001). MHV68 vGPCR is not constitutively active, however, it is activated by a wide array of cytokines containing ELR motifs to activate signaling pathways including nuclear factor kappa B (NF-κB) and Akt (Verzijl et al., 2004), and it can transform NIH3T3 cells in culture (Wakeling et al., 2001). The MHV68 v-cyclin promotes replication and reactivation *in vivo* (Upton and Speck, 2006), contributes to the immortalization of fetal liver B cells (Liang et al., 2011) and drives lymphomagenesis (van Dyk et al., 1999).

While many proteins involved in lytic replication are largely conserved amongst the gHVs, each gHV encodes unique genes. MHV68 contains both unique genes and non-coding RNAs (Bowden et al., 1997; Virgin et al., 1999; Reese et al., 2010; Zhu et al., 2010). The MHV68 genome encodes 14 unique M genes that are located throughout the viral genome, many have immunomodulatory functions that promote chronic infection (Evans et al., 2008; Siegel et al., 2008; Hughes et al., 2011). The left end of MHV68 encodes eight non-coding RNAs termed TMERS, viral tRNA-like molecules embedded with single or multiple 3’ miRNAs, that are expressed during latency and productive infection (Bogerd et al., 2010; Feldman et al., 2014). Recent work has uncovered a role for the viral TMERs in viral dissemination and latency establishment in wild-type (WT) mice, as well as pneumonia in an immunocompromised host (Feldman et al., 2014, 2016; Diebel et al., 2015). Specific targets for the miRNAs and the function of the tRNA-like molecules have not been determined.

### Pathogenic Features Common with Primate and Human Gammaherpesviruses

Murine gammaherpesvirus 68 also shares pathogenic similarities with both the primate and human gHVs (**Table 1**). MHV68 undergoes a subclinical period of acute replication upon infection of the nasal or respiratory mucosal tissue (Rajcani et al., 1985; Sunil-Chandra et al., 1992b; Milho et al., 2012). Productive lytic infection in the lung causes localized tissue damage that undergoes normal repair (Ehtisham et al., 1993). The initial infection is cleared from the lungs by 12 days post infection in BALB/c and C57BL/6 mice (Sunil-Chandra et al., 1992b; Cardin et al., 1996), then trafficked by B cells to the spleen (Usherwood et al., 1996c). MHV68 infection is associated with an expansion of lymphocyte populations that drives an infectious mononucleosis-like response marked by enlarged lymph nodes and splenomegaly (Usherwood et al., 1996a; Tripp et al., 1997; Flano et al., 2002b).

Upon gaining access to the spleen, MHV68 establishes latency in germinal center B cells (Collins et al., 2009). Levels of latency in the spleen contract by 3–4 weeks post-infection, and the predominant reservoir of viral latency shifts to the memory B cells as found for EBV (Miyashita et al., 1995, 1997; Babcock et al., 1998; Flano et al., 2002a; Willer and Speck, 2003). Immune competent animals typically control latent infection, but long-term studies reported a higher incidence of lymphomas between 6 months and 3 years post infection compared to uninfected mice (Sunil-Chandra et al., 1994). The latently infected B lymphoma cell line, S11, was derived from long-term MHV68-infected mice that spontaneously developed lymphoproliferative disease (Usherwood et al., 1996b). Additional immunosuppression via cyclosporine A administration, the loss of T cells or interferon gamma signaling increases the prevalence of lymphoproliferative disease, mirroring the development of gHV-associated malignancies in immunosuppressed humans (Sunil-Chandra et al., 1994; Tarakanova et al., 2005, 2008; Lee et al., 2009). In addition, MHV68 can drive fetal liver B cell immortalization and latency programs are associated with the development of B cell lymphomas in mice (Liang et al., 2011).

### Genetic Manipulation of Virus and Host Determinants

The MHV68 pathogenesis system allows for the mutation of host or viral genes, enabling *in vivo* analysis of determinants of viral pathogenesis. The MHV68 genome is cloned as a bacterial artificial chromosome system (BAC), allowing for straightforward mutagenesis of the viral genome in *E. coli* (Adler et al., 2000; Tischer et al., 2010). To better track the course of infection *in vivo*, several reporter viruses have been generated. These include a constitutively expressed YFP, a lytic promoter-driven luciferase marker, or direct reporter fusion with the major latency protein LANA (Hwang et al., 2008; Collins et al., 2009; Nealy et al., 2010). Likewise, the mouse system offers a wide array of germline and inducible knockouts of host genes to identify host determinants of infection and to facilitate a better understanding of the virus–host interactions that occur during multiple stages of infection. Coupling a virus with a reporter gene flanked by loxP sites with transgenic mice that express Cre in specific cell types has been applied to track the cells that MHV68 infects en route to the latency reservoir (Gaspar et al., 2011).

In addition, the accessibility of lytic and latent tissue culture models and the ability of MHV68 to replicate to high titer in tissue culture facilitate mechanistic studies *in vitro*. MHV68 replicates in murine fibroblasts, endothelial, epithelial, dendritic cells, and macrophages, with a single round of lytic replication lasting 12–18 h (Speck and Ganem, 2010). B cells can be *de novo* infected *in vitro*, albeit with low efficiency, and latent B cell lines have been established (Usherwood et al., 1996b; Forrest and Speck, 2008; Liang et al., 2011). Together, these systems allow for examination of multiple aspects of the viral life cycle *in vitro*.

## Course of MHV68 Infection in Mice

### Routes of Transmission

Epstein–Barr virus and KSHV are primarily contracted through the oral mucosa, and initial infection is often subclinical. EBV infection can lead to acute infectious mononucleosis (AIM), especially when contracted in adolescence or early adulthood (Hoagland, 1964). AIM patients generally exhibit symptoms many weeks after infection; thus, the initial kinetics of EBV replication in the oral epithelium and latency establishment of B cells are not well-understood (Sixbey et al., 1984; Dunmire et al., 2015). Natural transmission of MHV68 between animals is not clearly defined. MHV68 can readily infect the nasal and respiratory mucosa upon intranasal infection (Tan et al., 2014), but it is unclear if this occurs in the wild. MHV68 and the closely related MHV72 may undergo vertical transfer from mother to pup, as viral DNA can be detected in the breast milk of infected dams and stomachs of the pups (Raslova et al., 2001; Stiglincova et al., 2011). However, bone fide vertical transmission was not observed since latency was not established in the pups (Francois et al., 2013). Co-housing of latently infected animals with naïve same-sex animals is insufficient for horizontal transmission (Francois et al., 2013).

Recent studies support a sexual mode of transmission for MHV68. Epidemiologic studies of wild mice found the highest prevalence of infection in the heaviest, most sexually active males (Telfer et al., 2007). High titers of virus inoculum applied to the vagina of mice led to intranasal infection of naïve mice (Milho et al., 2012). Intermittent virus replication has been reported in the genitalia of intranasally infected mice during latent infection (Francois et al., 2013). Indeed, MHV68 was transmitted from a female reactivating at the genitalia to a naïve male upon sexual contact (Francois et al., 2013).

### Virus Colonization of the Host

**FIGURE 1:**
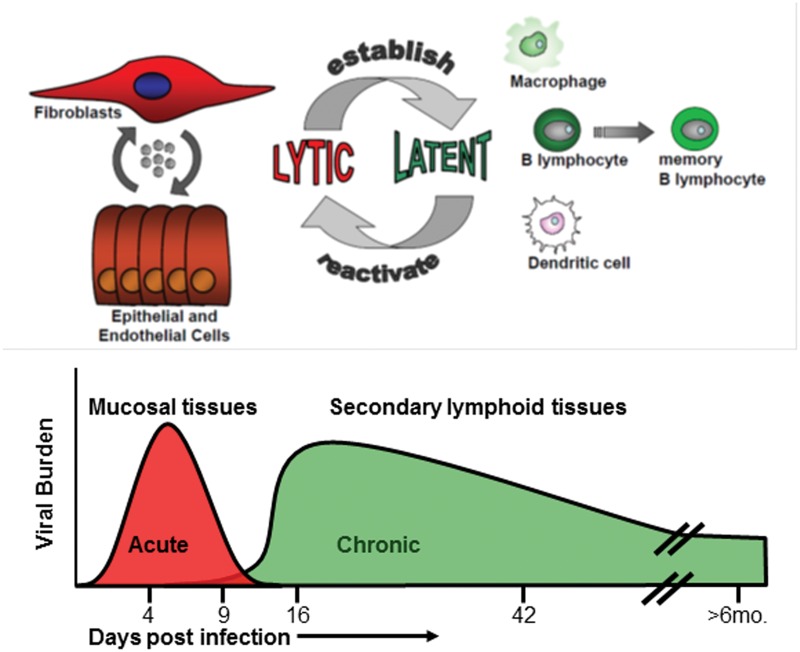
**Course of an MHV68 infection in mice.** Acute lytic replication occurs in the lung (intranasal infection) or in the spleen (intraperitoneal infection). Ongoing replication is cleared typically by 12 days. The peak of latency is measured around 16 days post-infection in secondary lymphoid tissues such as the spleen. Maintenance of latency is typically evaluated 42 days post-infection. B cells are the predominant latency reservoir, followed by macrophages, dendritic cells, and endothelial cells. Lymphomas arise at late stages in immunocompromised animals.

Infection of mice with MHV68 has given us insight into how gHVs ultimately gain access to the B cell memory reservoir. Viruses that mark infected cells have been coupled with cell-specific knock-out or ablation approaches to reveal a complex series of events that culminate in colonization of the spleen (Gaspar et al., 2011; Frederico et al., 2012, 2014a,b, 2015). The virus likely undergoes sequential infection of multiple cell types during transit from the site of initial infection- nasal or lung tissue in laboratory settings- to the draining lymph nodes and the spleen (**Figure 1**). MHV68 infection *in vivo* does not result in high levels of cell-free virus (Sunil-Chandra et al., 1992a), and the virus depends on cell-to-cell spread for efficient propagation (May et al., 2005). Hematogenous dissemination is likely involved; deletion of MHV68 non-coding RNA TMER4 is sufficient to reduce viral burden in peripheral leukocytes and impair infection of the spleen and peritoneal cavity (Feldman et al., 2016). In the absence of B cells, seeding of the secondary lymphoid tissues is significantly decreased, and mice do not develop an infectious mononucleosis-like syndrome (Usherwood et al., 1996c; Chao et al., 2015). Even in the absence of B cells, latency is detected in the spleen at late times post infection, suggesting alternative routes that allow access to distal reservoirs of latency (Weck et al., 1996). MHV68 latency in non-B cells such as macrophages requires the function of v-cyclin to drive reactivation and maintain the viral reservoir (van Dyk et al., 2003).

### Upper Respiratory Tract Infection

Intranasal infection without the use of anesthetic delivers the viral inoculum into the upper airways (Tan et al., 2014). Upon inoculation of the upper respiratory tract infection, MHV68 first infects the neuroepithelium of the rostral nasal cavity (Frederico et al., 2012; Milho et al., 2012). Infection of the neuroepithelium is dependent on viral heparin sulfate-binding glycoproteins (Milho et al., 2012). From here, infection progresses to the draining superficial cervical lymph nodes via dendritic cells, after which the virus transits to the spleen (Milho et al., 2009; Gaspar et al., 2011). Upper respiratory tract infection leads to similar levels of splenic latency in the spleen as that obtained from deeper lung infection; however, there is a kinetic delay in viral colonization of the spleen (Milho et al., 2009). Subcapsular sinus macrophages that filter the afferent lymph nodes become infected by MHV68, yet produce few infectious progeny virions. Interestingly, their depletion increased splenic B cell infection, and led the authors to propose that this cell type may represent a bottleneck to dissemination (Frederico et al., 2015).

### Lower Respiratory Tract Infection

Murine gammaherpesvirus 68 pathogenesis studies typically report the inoculation of mice by autonomic deep inhalation occurring after light anesthesia. A large bolus of liquid is inhaled deep into the lung, infecting both the upper respiratory tract as well as the lung epithelium, but neither the trachea nor bronchi (Milho et al., 2009). Intranasal inoculation leads to viral attachment to type 1 alveolar epithelial cells and initial infection of alveolar macrophages (Lawler et al., 2015), which collect particulate from the alveolar lumen (Geiser, 2002). From these macrophages, infection spreads to type 1 alveolar epithelial cells (Sunil-Chandra et al., 1992a; Nash and Sunil-Chandra, 1994; Milho et al., 2009; Lawler et al., 2015). Viral titer increases in the lungs, peaking at 7 dpi, before being cleared by 12 dpi (Ehtisham et al., 1993), with CD8+ T cells playing a central role in this process (Weck et al., 1996; Doherty et al., 2001). Interestingly, despite the significant expansion of virus in the lungs and evidence of inflammatory infiltrate, tissue repair largely restores lung morphology (Sunil-Chandra et al., 1992a; Ehtisham et al., 1993; Dutia et al., 1997). There is significant infection of macrophages in the lungs by this method, and the virus primarily drains to the mediastinal lymph node via a dendritic cell-independent route (Gaspar et al., 2011). The virus then transits to the spleen in a B cell dependent manner (Usherwood et al., 1996c).

### Role of Productive Replication in Dissemination and Latency

The influence of acute replication defects in the lung on the establishment of latency after intranasal inoculation is well documented. MHV68 mutants lacking genes necessary for productive replication or DNA replication fail to establish WT levels of latency in the spleens of infected mice after intranasal inoculation (Coleman et al., 2003; Flano et al., 2005; Moser et al., 2006; Tibbetts et al., 2006; Gill et al., 2009, 2010; Milho et al., 2011; Minkah et al., 2015). Splenic latency could be partially or fully restored after altering the route of infection or dramatically increasing virus dose (Coleman et al., 2003; Flano et al., 2005; Gill et al., 2009, 2010; Milho et al., 2011; Minkah et al., 2015). Together these observations imply a critical role for lytic replication in seeding the latent splenic reservoir after intranasal inoculation. For many mutants, it remains to be determined if phenotypes represent an inability to replicate in mucosal tissues at sites of entry or represent an inability to efficiently infect immune cells critical for the dissemination of MHV68 to the B cells in the spleen. Alternatively, some degree of ‘lytic’ gene expression might be required to establish a latent gene expression in a particular cell-type.

### Seeding of the Germinal Center

The route of MHV68 to the germinal center B cell likely involves multiple cellular transitions (Gaspar et al., 2011; Frederico et al., 2012, 2014a, 2015). Blood entering the spleen in a central arteriole first passes through the macrophage-rich marginal sinuses. These macrophages are the first splenocytes to become infected by MHV68, providing an entryway into the white pulp and expanding lymphocyte reservoir (Frederico et al., 2014a). Infection of these marginal zone macrophages leads to lytic infection and the production of virions that are better suited to infect B cells (Frederico et al., 2012). These virions infect adjacent marginal zone B cells that carry the virus to the emerging germinal centers (Frederico et al., 2014a). Marginal zone B cells associate with and deposit virions onto the follicular dendritic cells (Frederico et al., 2014a). The dendritic cells then seed the expanding population of germinal center B cells, where latent infection is detected at the highest frequency (Sunil-Chandra et al., 1992b). Although the germinal center B cell response subsides after the 14–18 dpi peak, latency is maintained in germinal center B cells as well as memory B cells at late times after infection (Flano et al., 2002a; Willer and Speck, 2003; Nealy et al., 2010). T follicular helper cells play a critical role by supporting germinal center formation and infected B cell expansion through the secretion of the pleiotropic cytokine IL-21 (Collins and Speck, 2014, 2015).

ORF50-null viruses that lack the key lytic gene transactivator, replication and transcription activator (RTA), are impaired for splenic latency establishment (Moser et al., 2006; Frederico et al., 2014a). An ORF50-null mutant failed to seed the marginal zone B cells as visualized by fluorescence analysis of infected spleen sections (Frederico et al., 2014a). An ORF27-stop virus, lacking the gp48 glycoprotein, was only slightly attenuated for lytic replication in cell culture but was greatly impaired for latency establishment upon intraperitoneal inoculation (May et al., 2005). As with the ORF50-mutant, the ORF27 mutant remained in the marginal zone macrophage cells and did not seed B cells in the white pulp (Frederico et al., 2014a). Taken together, defects in lytic replication and intercellular spread impact latency establishment in the B cell reservoir, even when the virus is administered by a more direct route. Examination of trafficking defects in additional mutants are needed to validate and extend this developing model for MHV68 dissemination.

While these trafficking studies provide insight into the pathogenesis of gHV infection, it is important to note that there are differences in the splenic architecture of mice and humans. In humans, the marginal sinuses and associated macrophages reside more proximal to the follicle in a region called the perifollicular zone, rather than in the more distal marginal zone where the marginal zone B cells reside in the mouse (Steiniger et al., 1997). While the path to the germinal center may differ slightly between species, MHV68 studies suggest that access to the memory B cell involves transition through multiple cell types, providing additional strategies to interfere with chronic gHV infection.

### Maintenance of Latency

Latency is established in dendritic cells, macrophages, epithelial, and endothelial cells (Weck et al., 1999b; Flano et al., 2000; Suarez and van Dyk, 2008), and many B cell subsets (Nealy et al., 2010; Coleman et al., 2014; Feldman et al., 2016). The peak of viral latency occurs around 16 days post-infection, but viral genomes are detectable at late times during chronic infection (**Figure 1**). Although MHV68 initially gains access to the germinal center B cell reservoir, it is predominantly found in immunoglobulin isotype class-switched memory B cells during long-term latency (Flano et al., 2002a; Marques et al., 2003; Willer and Speck, 2003). Access to, and establishment of, latency in the memory B cell reservoir provides the gHV with a long-lived cellular reservoir that can reside in lymphoid tissue or circulate through the body (Giesecke et al., 2014). However, memory B cells likely undergo homeostatic proliferation and terminal differentiation into plasma cells upon receptor engagement, for instance in the context of co-infections.

During infection, MHV68 also infects pro-B, pre-B, and immature B cells in the bone marrow and is detected in immature and transitional B cells during chronic infection (Coleman et al., 2010). Depletion of developing B cells reduces latency in the mature B cells (Coleman et al., 2014). Normal B cell maturation is dependent on limited survival signals and deletion of self-reactive cells. Interestingly, MHV68 vBcl-2 functions to prevent apoptosis following activation of the BCR (Coleman et al., 2014), perhaps allowing MHV68 infected cells to bypass normal B cell selection checkpoints.

### Reactivation from Latency

Latent cells infected with gHVs can be reactivated using chemical or biological agents *in vitro*, but the stimuli for reactivation in the immune competent host is poorly understood (Murata, 2014). MHV68 has been used to further evaluate reactivation *in vivo.* A critical event in induction of reactivation of the gHVs is the terminal differentiation of the B cell into a plasma cell (Laichalk and Thorley-Lawson, 2005; Bhende et al., 2007; Sun and Thorley-Lawson, 2007; Wilson et al., 2007; Yu et al., 2007; Siegel et al., 2010). This phenomenon appears to be driven by the transcription factors IRF-4 and XBP-1, which are expressed upon B cell maturation into plasma cells (Crawford and Ando, 1986; Laichalk and Thorley-Lawson, 2005; Matthias and Rolink, 2005; Yu et al., 2007). The human gHVs are dependent on XBP-1 for reactivation (Laichalk and Thorley-Lawson, 2005; Bhende et al., 2007; Sun and Thorley-Lawson, 2007; Wilson et al., 2007; Yu et al., 2007), while MHV68 requires IRF-4 (Matar et al., 2014). MHV68 M2 is a latency-associated gene (Virgin et al., 1999) that promotes B cells differentiation into a plasma cell-like phenotype (Liang et al., 2009). Deletion of the plasma cell lineage causes a reduction in reactivation as well as a contraction of the latent reservoir during long-term infection (Siegel et al., 2010). *In vivo* injection of TLR agonists into a latently infected animal drives reactivation, and results in a higher latent viral load (Gargano et al., 2009). Stimulation of either CD40 or TLR9 can also drive reactivation in cell culture (Moser et al., 2005; Ptaschinski et al., 2010).

A latent gHV is likely influenced by the activation status of the immune compartment and cytokines in the host. Co-infection of MHV68 infected mice with the pathogenic helminths *H. polygyrus* or *S. mansoni* induces reactivation (Reese et al., 2014). The mechanism appears to be IL-4 induction by helminth infection that reduces IFNγ levels, a cytokine that represses the expression of the lytic transactivator RTA to promote the maintenance of viral latency (Reese et al., 2014). Recently, impairment of autophagy in macrophage cells was found to suppress reactivation by increasing inflammation and T cell activation to drive IFN-γ production (Park et al., 2016).

### B Cell-Mediated Control of Infection

In addition to serving as the primary reservoir of latency, B cells contribute to immune control of gHV infection. Studies in B cell-deficient mice reveal high levels of viral latency and reactivation in the peritoneum (Weck et al., 1999a), but the lack of B cells also impairs the development of Vβ4 T cells, which secrete IFNγ and control viral reactivation (Weck et al., 1996; Flano et al., 1999). Passive transfer of immune sera into B cell deficient μMT mice reduces the establishment of viral latency (Gangappa et al., 2002). Infection of mice lacking CD28, a costimulatory molecule necessary for T cell support of germinal center B cells, reveals only slightly reduced latency establishment and normal antibody response (Kim et al., 2002). However, this antibody response was poorly neutralizing and rapidly decreased over time. Depletion of T cells from CD28^-/-^ mice led to recrudescence of the virus that could be inhibited by passive transfer of immune sera, indicating that humoral immunity contributes to the control of latent infection (Kim et al., 2002). In addition, immunization of mice with a reactivation-deficient virus generates immune sera that can prevent superinfection by a WT virus (Tibbetts et al., 2003). Interestingly, neutralizing antibody can prevent infection of cell types that do not express Fc receptors, but increases infection of cell types expressing Fc receptors (Rosa et al., 2007). To tease apart the contributions of B cell and T cell interactions from the generation of anti-viral antibody, McClellan et al. (2006) infected mice with a germline rearrangement of an antibody variable region such that all B cells to produce antibodies specific to hen egg lysozyme. While virus-specific antibody was not necessary for control of latent infection, depletion of CD8+ T cells increased latency and reactivation in the peritoneum and depletion of either CD4+ or CD8+ caused increased latency and reactivation in the spleen (McClellan et al., 2006).

### T Cell-Mediated Control of Infection

Numerous studies have identified roles for both CD4+ and CD8+ T cells in clearance of acute lytic infection in the lungs (Ehtisham et al., 1993; Cardin et al., 1996; Stevenson et al., 1999b; Molloy et al., 2011). During long-term infection, loss of CD4+ T cells compromises immune control, leading to recrudescence in the lungs, progressive pathology, and death (Cardin et al., 1996; Molloy et al., 2011). Cytotoxic CD8+ T cells also play an important role in control of early gHV infection; however, their loss leads to a much different phenotype than that of CD4+ T cell-deficient animals. In Balb/c mice, depletion of CD8+ T cells after lung infection causes persistent lytic infection in the lung as well as the spleen and is accompanied by significant clinical pathology and increased necrosis of the lung (Ehtisham et al., 1993). This phenomenon is strain-dependent, as C57BL/6 mice depleted of CD8+ T cells suffer delayed clearance from the lung accompanied by increased viral latency in the spleen (Stevenson et al., 1999b). In both backgrounds, simultaneous depletion of CD4+ and CD8+ T cells leads to persistent virus replication and death (Ehtisham et al., 1993; Stevenson et al., 1999b).

While T cells play an important role in initial clearance of viral infection, they are also crucial for control of latent infection. It is telling that much of the burden of human disease caused by the gHVs occurs during T cell immunosuppression (Malnati et al., 2003; Thompson and Kurzrock, 2004; Cesarman, 2014). CD8+ T cells specific to MHV68 are observed in the infected mice as soon as 1 week after infection and are critical for immune surveillance and control of gHV latency (Stevenson and Doherty, 1998; Stevenson et al., 1999a; Gredmark-Russ et al., 2008). This response consists of multiple immunodominant and subdominant T cells that shift over time (Stevenson et al., 1999a; Freeman et al., 2010). During latency, CD8+ T cells serve to control viral reactivation at two important sites of latency: the spleen and the peritoneum (Tibbetts et al., 2002). Latency establishment occurs normally in the absence of CD4+ T cells (Stevenson et al., 1999b). Unlike CD8+ T cell KO animals, CD4+ T cell KO mice fail to contract the latent reservoir in the spleen and suffer a progressive increase in reactivation in the lungs, eventually leading to death (Cardin et al., 1996). This defect can be rescued with anti-CD40 antibody treatment in a CD8+ T cell dependent manner, indicating that CD4+ T cells provide important costimulatory signals to CD8+ T cells (Sarawar et al., 2001; Dias et al., 2010).

## The NF-κB Signaling Pathway

Despite the importance of latency in the viral life cycle, the mechanisms that drive latency establishment in some cells types but not others, and the cellular signals that maintain latency or initiate reactivation are poorly understood. Better understanding of the host signaling pathways that contribute to the establishment and maintenance of a latent infection is critical for the development of therapeutics against infection and virus-associated malignancies. The NF-κB pathway is a signaling process that involves upstream phosphorylation events that culminate in the proteasomal processing of regulatory proteins that enable translocation of NF-κB transcription factors into the nucleus. NF-κB signaling plays a critical role in many cellular processes, including cell survival and inflammatory signaling, as well as a central role in B cell maturation.

**FIGURE 2:**
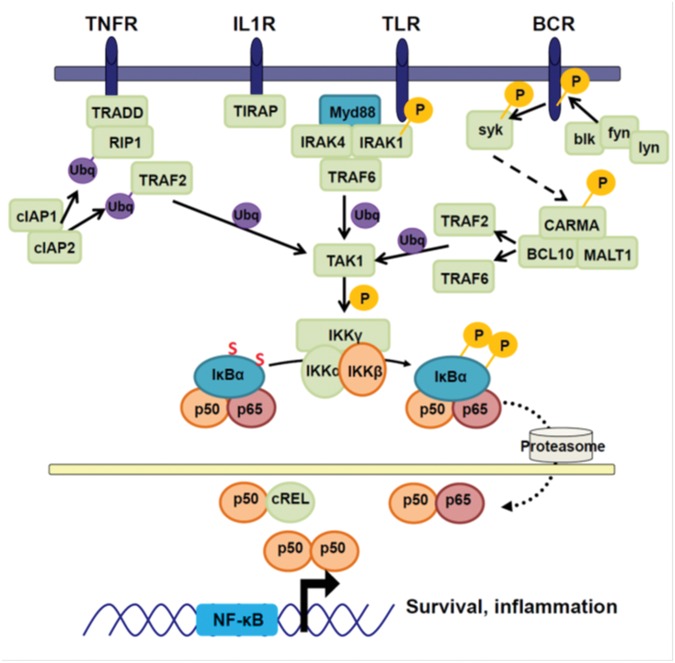
**Canonical NF-κB signaling.** Canonical NF-κB signaling is activated by the binding of extracellular ligands. Each receptor family differs in the upstream signaling molecules used to mediate signaling, but they share activation of TRAF2 and TRAF6. The TRAFs ubiquitinate TAK1, causing TAK1 to be activated and associate with IKKγ. TAK1 can then phosphorylate IKKβ, which in turn phosphorylates IκBα. Phosphorylated IκBα is degraded by the proteasome, releasing the canonical transcription factors p65, p50, and cREL. These translocate into the nucleus and activate pro-survival and pro-inflammatory genes.

**FIGURE 3:**
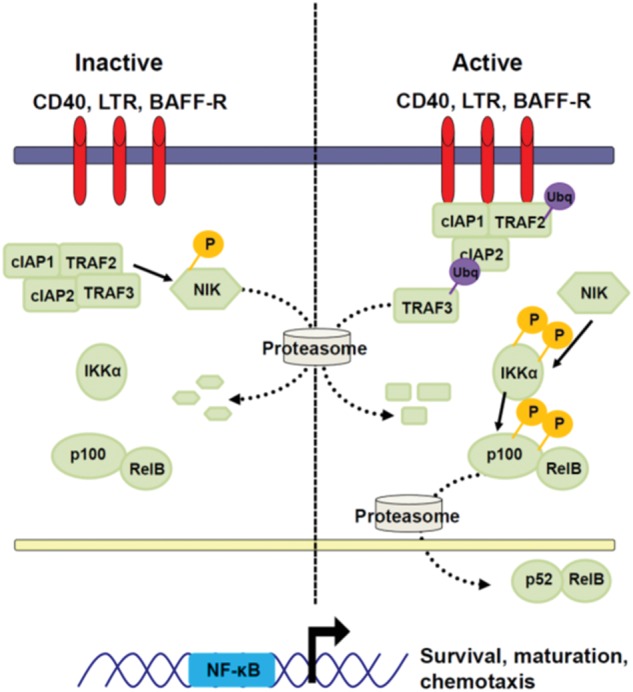
**Non-canonical NF-κB signaling.** Non-canonical NF-κB signaling is initiated by the binding of cell surface receptors to their ligands. Trimerization of the receptors recruits TRAF2, cIAP1, and cIAP2 to the receptor. This changes the specificity of cIAP1/2 ubiquitin ligation from NIK to TRAF3. TRAF3 is ubiquitinated by this complex and degraded by the proteasome. The absence of TRAF3 prevents degradation of NIK, leading to its accumulation. NIK can phosphorylate IKKα. Activated IKKα phosphorylates p100, inducing its partial degradation by the proteasome. The processed p100 subunit, p52, associates with RelB and translocates into the nucleus where it mediates transcription of pro-survival genes.

In resting cells, NF-κB complexes are retained in the cytoplasm by inhibitors of NF-κB (IκBs). IκBs are phosphorylated by IκB kinase (IKK) complexes resulting in activation of canonical or non-canonical NF-κB pathways. The NF-κB family consists of five subunits, p65 (RelA) RelB, cRel, p50/p105 (NF-κB1), and p52/p100 (NF-κB2) that can form homo- or hetero-dimers. Depending on the composition of the dimers and other variables such as post-translational modification and interactions with other factors, NF-κB dimers can either induce or repress gene expression through direct binding to DNA sequences in the regulatory region of target genes and also through interactions with other factors (Oeckinghaus and Ghosh, 2009). NF-κB signaling consists of two distinct pathways; the IKKβ-dependent canonical pathway (**Figure 2**), and the IKKα-dependent non-canonical pathway (**Figure 3**).

### Canonical NF-κB Signaling

The canonical pathway is activated by the IKKα/β/γ complex and results in IKKβ-mediated phosphorylation and ubiquitin-mediated proteosomal processing of IκBα proteins, which sequester NF-κB subunits p50/p65 in the cytoplasm. Degradation of IκBα unmasks the nuclear localization signals of the subunits, permitting their nuclear translocation. Canonical subunits have preferential binding partners, p65 with c-Rel or p50, and p50 dimerizing with itself (Vallabhapurapu and Karin, 2009). These dimers have subtly different target sequences, and can associate with other transcription factors to control their target genes (Urban and Baeuerle, 1991; Kunsch et al., 1992; Perkins et al., 1992; Bonizzi et al., 2004). The canonical NF-κB signaling pathway serves a broad range of functions within the body, contributing to immune activation in response to cytokines and pro-inflammatory signaling in response to pathogen detection (Li et al., 2002). These cell surface receptors include: the IL-1R and the TNFR family of receptors which bind cytokines, TLRs that recognize pathogen-associated molecular patterns, and the B and T cell receptors, which provide pro-survival signals. Each receptor transduces its signal with an overlapping set of effector molecules that are summarized in **Figure 2**.

### Non-canonical NF-κB Signaling

Non-canonical/alternative NF-κB signaling mediates organo genesis, cell maturation, and pro-survival signaling. This pathway is activated by specific members of the TNFR family, such as CD40, BAFFR, and TNFRSF3 (LTβR; Matsushima et al., 2001; Sun, 2012) (**Figure 3**). In contrast to the canonical pathway receptors, activated receptors of the non-canonical NF-κB pathway directly associate with TRAF proteins (Pullen et al., 1999; Hildebrand et al., 2010) through multiple TRAF binding sites on their intracellular tails (Pullen et al., 1998). In resting cells, NF-κB-inducing kinase (NIK), which is the major IKKα-activating kinase in cells, is constitutively active, but is present at very low levels due to its constitutive degradation by cIAP1/2 (Varfolomeev et al., 2007), dependent on TRAF3 and TRAF2 (Liao et al., 2004; Gardam et al., 2008). Relief from constitutive degradation by receptor engagement and TRAF3 degradation allows for activated NIK to accumulate (Dejardin et al., 2002; Gardam et al., 2008). This accumulation drives the phosphorylation of IKKα on two serines in its activation loop (Regnier et al., 1997; Lin et al., 1998; Ling et al., 1998) and licenses IKKα to phosphorylate p100 (Senftleben et al., 2001). Full-length p100 can act similarly to IκBα, sequestering NF-κB subunits in the cytoplasm. Upon phosphorylation, p100 undergoes partial proteolysis to p52 that can associate with the NF-κB transcription factor RelB and translocate to the nucleus to initiate gene transcription (Xiao et al., 2001; Solan et al., 2002; Bonizzi et al., 2004). Activated IKKα also serves to negatively regulate the non-canonical pathway signaling through phosphorylation and destabilization of NIK (Razani et al., 2010).

### Crosstalk between Canonical and Non-canonical NF-κB Signaling

Extensive research has defined the signaling pathways for each arm of NF-κB signaling, as well as numerous levels of crosstalk between these two arms. Full-length p100 may sequester canonical subunit dimers in addition to RelB in the cytoplasm, interfering with canonical signaling (Basak et al., 2007). Canonical NF-κB signaling can influence non-canonical pathway signaling through the induction of RelB and p100 transcription (Dejardin et al., 2002; Basak et al., 2008). NF-κB1 subunit p50 may compete with p52 for binding to RelB, (Basak et al., 2008), and p65 can bind p100 and induce its degradation, leading to the formation of p52/p65 heterodimers (Hoffmann et al., 2003). The use of common upstream signaling molecules and the formation of NF-κB heterodimers likely contributes to extensive overlap in target genes between canonical and non-canonical NF-κB pathways (Li et al., 2002).

### The Role of NF-κB Signaling in B Cell Development

**FIGURE 4:**
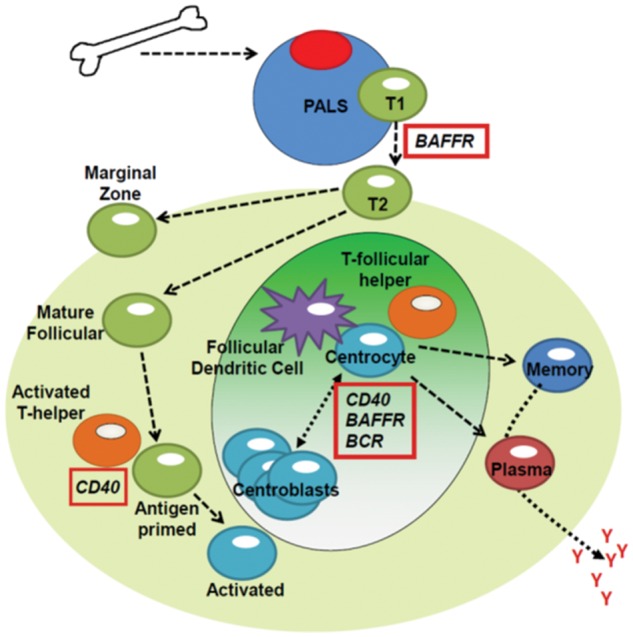
**Role of NF-κB signaling in B cell maturation and differentiation.** B cells exit the bone marrow and home to secondary lymphoid organs. The B cell exits from the arteriole into the peripheral arteriole lymphoid sheath (PALS) as a T1 B cell. Here, the B cell requires BAFF signaling to survive and mature to a T2 B cell. The T2 B cell can mature to a marginal zone B cell or follicular B cell. The mature follicular B cell can be activated by the detection of cognate antigen and CD40 costimulation. The activated B cell enters the germinal center, where it competes for antigen and costimulatory factors presented on follicular dendritic cells and produced by T follicular helper cells. Strong antigen binding and receipt of costimulatory NF-κB-dependent signaling through CD40 and BAFF allows the B cell to mature into a memory B cell or a plasma cell.

Nuclear factor-kappa B signaling pathways are utilized by B lymphoid cells, the primary latency reservoir for gHVs, to drive transcription of genes important for B cell maturation, responses to infection, proliferation, protection from apoptosis, and immunoglobulin isotype class switching (Pasparakis et al., 2006). B cells develop in the bone marrow where they undergo VDJ recombination and emerge with a functional heavy and light immunoglobulin chain (Ichii et al., 2014). The B cells emerge into the periphery as transitional 1 (T1) B cells, characterized as CD23^lo^ IgM^hi^ (Hsu et al., 2002), and home to secondary lymphoid organs (**Figure 4**). Successful entry to the periarteriolar lymphoid sheath of the spleen (Loder et al., 1999) exposes the nascent B cell to peripheral selection by BAFF signaling. BAFF is secreted by numerous cell types in the spleen, including myeloid, dendritic, and stromal cells (Nardelli et al., 2001; Litinskiy et al., 2002; Gorelik et al., 2003). BAFF binds primarily to the BAFF receptor (BAFFR) on B cells, inducing pro-survival Bcl-2 family members (Claudio et al., 2002) and down-regulating apoptotic genes (Craxton et al., 2005) through non-canonical NF-κB, Akt, and ERK signaling in an IKKα-dependent manner (Claudio et al., 2002; Patke et al., 2006; Otipoby et al., 2008; Woodland et al., 2008; De Silva et al., 2016). BAFF, BAFFR, or IKKα- knockout cells mature and exit the bone marrow but undergo apoptosis upon entry to the spleen, reducing the transitional 2 (CD23^hi^ IgM^hi^) and mature B cells by up to 95% (Lentz et al., 1996; Kaisho et al., 2001; Senftleben et al., 2001; Hsu et al., 2002). Conversely, transgenic expression of BAFF in mice drastically expands the transitional and marginal zone B cell reservoirs (Batten et al., 2000). Upon maturation past the T1 phase into the T2 phase, the nascent B cell relocates to the splenic follicle where it undergoes BCR-dependent selection (Loder et al., 1999) mediated by the NF-κB signaling pathway (Lucas et al., 2001; Wang et al., 2001; Shinohara et al., 2005). Successful activation licenses the T2 B cell to become a marginal zone or naïve mature follicular B cell (Loder et al., 1999).

The next step of B cell maturation occurs upon detection of a cognate antigen for the BCR, accompanied by CD40 co-signaling, leading to the formation of the germinal center (Goetz and Baldwin, 2008). CD40 signals through both IKKα and IKKβ (Kosaka et al., 1999). Mice lacking CD40 fail to form germinal centers (Goetz and Baldwin, 2008). Germinal centers can form from the small population of mature B cells in BAFF^-/-^ mice during infection, although these are short-lived (Rahman et al., 2003; Vora et al., 2003). Both of the downstream IKKs are necessary for passage of the B cell through the germinal center of mice (Goetz and Baldwin, 2008). A dominant negative knock-in of IKKα (IKKαSSAA) abrogates germinal center formation (Kaisho et al., 2001; Mills et al., 2007), while conditional deletion of IKKβ drastically impairs global B cell survival (Pasparakis et al., 2002). It has been recently shown that conditional deletion of NIK after passage of the T1 checkpoint impairs the ability of B cells to enter the germinal center, further confirming the necessity of non-canonical signaling in the germinal center reaction (Brightbill et al., 2015).

## Disruption of the NF-κB Signaling Pathways Alters MHV68 Pathogenesis

During the course of infection, the gHVs traverse numerous cell types before gaining access to germinal center B cells, and ultimately memory B cells. However, the signaling events that determine the lytic or latent course of infection are not well understood. The NF-κB signaling pathway is activated during B cell maturation and differentiation. Numerous gHV proteins can activate or impair signaling through the expression of viral proteins and miRNAs. While these viral molecules are important for latency and infected cell survival in culture, the role of NF-κB signaling in the gHV lifecycle of the host is not well-defined. The genetic tractability of the MHV68 pathogenesis system has enabled the *in vivo* role of many NF-κB signaling components to be examined (**Table 2**).

**Table 2:** *In vivo* studies of the role of NF-κB signaling on MHV68 pathogenesis.

Gene	Knockout	Immune defects	Route/Dose	Impact on pathogenesis	Changes in host response	Reference
LTα	Germline	No lymph nodes	IN^a^/2 × 10^5,b^	↓ Clearance from lung	↑ Lung inflammation	Lee et al., 2000
				↑ Latency	↓ CD8+ T cell activation	
CD40	Germline	No germinal center formation	IN/1 × 10^3^	↑ Reactivation		Willer and Speck, 2005
				↑ Infection of naïve B cell		
				Recrudescence in lungs		
CD40	Mixed BM chimera		IN/4 × 10^2^	Equal latency establishment Loss of latency in CD40^-/-^ subset	Exclusion of CD40^-/-^ B cells from germinal center	Kim et al., 2003
BAFFR	Germline	↓ Mature B cells Impaired Ab^c^ production	IN/1 × 10^4^	↓ Latency at 30 dpi^d^	↓ Germinal centers	Frederico et al., 2014b
					↓ MHV68-specific Ab	
			IP^e^/1 × 10^5^	↑ Viral titer at 3 dpi,		
				↓ Infection from 8–30 dpi		
TLR2/9	Germline		IN/5 × 10^4^	↑ Viral load at 3 dpi		Sun et al., 2015
TLR3	Germline		IN/1 × 10^3^	None		Gargano et al., 2008
TLR7	Germline		IP/1 × 10^5^	None		Haas et al., 2014
TLR9	Germline		IP/1 × 10^5^	↑ Reactivation		
MyD88	Germline	↓ B cell activation	IN/1 × 10^3^	↓ Latency in spleen at 16 dpi	↓ B cell activation	Gargano et al., 2008
				No difference in latency at 90 dpi	↓ Germinal centers	
					↓ Class switching	
					Delayed ab production	
			IP/1 × 10^3^	↑ Reactivation in PECs		
				↓ Reactivation in spleen		
	Mixed BM ^f^ Chimera		IN/1 × 10^3^	↓ Latency	↓ MyD88^-/-^ germinal center entry	
IkBaM	Viral Transgene		IN/1 × 10^3^	↓ Latency establishment		Krug et al., 2007
				↓ Infected lung B cells		
			IP/1 × 10^3^	↓ Viral titer		
				↓ Latency		
p50	Germline	↓ B cell proliferation	IN/1 × 10^2^	↑ Viral titers in lung	↓ Antibody production	Krug et al., 2009
		↓ Ab levels		↓ Latency at 16 dpi	↓ Vβ4+ T cell	
		↓ Germinal centers		↑ Latency over time		
				Recrudescence in lungs		
	Mixed BM chimera	None	IN/1 × 10^2^	Normal lung titers	↓ p50^-/-^ germinal center B cell	
				↓ Latency in p50^-/-^ B cells		
				Recrudescence in lungs		


*^a^IN, intranasal infection under light anesthesia.*

*^b^Dose described as PFU per animal.*

*^c^Ab, antibody.*

*^d^dpi, days post infection.*

*^e^IP, intraperitoneal infection.*

*^f^BM, bone marrow.*

### Role of NF-κB Signaling during Acute Phase of Infection

Infection with a recombinant MHV68 expressing a dominant-negative IκBα that blocks canonical NF-κB subunit activity did not impact viral replication in fibroblasts or lung epithelial cells in culture, and did not alter levels of acute expansion in the lungs of infected mice (Krug et al., 2009, 2010). Likewise viral replication proceeds normally in cells lacking the p50 subunit (Krug et al., 2009). Thus, NF-κB subunits are dispensable for MHV68 replication (Krug et al., 2007, 2009, 2010). In contrast, overexpression of the NF-κB subunit p65 was reported to impair RTA activation and virus replication, and suggested that NF-κB signaling is deleterious to lytic gene expression (Brown et al., 2003a). As detailed in the section describing gHV modulation of NF-κB signaling below, recent studies have determined that the virus subverts upstream signaling events while impairing subunit activation to enhance lytic gene expression (Dong and Feng, 2011; Dong et al., 2012; Karijolich et al., 2015).

The NF-κB p50 subunit is activated during both lytic and latent infection *in vitro* (Krug et al., 2009). Germline deletion of the NF-κB1 subunit that is processed to p50 in mice leads to higher acute lung replication after intranasal infection (Krug et al., 2009). The p50^-/-^ mice also exhibited reduced B and T cell responses upon MHV68 infection. The increase in lytic replication was rescued in bone-marrow chimeric mice generated from WT and p50^-/-^ donors, indicating the increased viral replication is not a cell-intrinsic effect, but rather due to impaired immune control (Krug et al., 2009). Mice lacking lymphotoxin alpha (LTα), a homolog of TNFα that signals through the canonical NF-κB pathway (Medvedev et al., 1996), lack lymph nodes and germinal centers in the spleen (Lee et al., 2000). Upon infection of LTα^-/-^ mice, viral titers in the lung were increased and clearance was delayed, and mutant mice had a transient increase in virus replication upon colonization of the spleen as compared to WT mice (Lee et al., 2000). The whole body knock-outs emphasize the role of the immune response in control of acute expansion that can impact dissemination to the spleen and long-term immune control of gHV infection.

Interestingly, interferon-γ receptor knockout mice typically succumb to MHV68 infection via viral persistence that drives fibrosis of organs including the lung (Mora et al., 2005). Lung fibrosis and mortality in the IFNγR^-/-^ mice was prevented by overexpression of a dominant-negative IκBα from a recombinant MHV68 (Krug et al., 2010). The inhibition of NF-κB subunit function both reduced viral load and the expression of the inflammatory chemokines MCP-1 and CXCL12 in the infected tissues (Krug et al., 2010). Further investigation is required to determine how NF-κB signaling in the infected tissue contributes to inflammatory cytokines and immune activation during infection.

### Role of NF-κB Signaling in Latency Reservoirs of the Host

Numerous *in vivo* investigations have demonstrated that disruption of NF-κB signaling can impair MHV68 latency establishment in B cells and alter long-term immune control of viral latency (**Table 2**). LTα^-/-^ mice exhibited increased levels of latency in the spleen that are accompanied by a reduction in T cell activation (Lee et al., 2000). The deletion of CD40, a cell surface molecule that activates canonical and non-canonical NF-κB signaling pathways upon CD40L engagement (Kosaka et al., 1999), prevented germinal center formation and T-dependent class switching (Kawabe et al., 1994). Although latency was established in class-switched B cells in CD40^-/-^ mice at levels comparable to WT mice, reactivation was increased in the spleen and recrudescence was observed in the lungs of the CD40^-/-^ mice (Batten et al., 2000; Willer and Speck, 2005; Kalled, 2006; Frederico et al., 2014b). The absence of MyD88, a molecule that acts downstream of numerous canonical pathway receptors, led to a defect in germinal center formation and a reduction of latency establishment early after infection with MHV68. However, latency ‘recovered’ to WT levels by late times post-infection (Gargano et al., 2008). Interestingly, infection of MyD88^-/-^ mice with the IκBαM-transgenic virus led to an even greater defect in latency establishment, suggesting that MyD88 has additional NF-κB subunit-independent effects on the latency reservoir (Gargano et al., 2008). Germline deletion of a single NF-κB subunit, p50, led to a significant reduction in latency establishment (Krug et al., 2009). The defects in B and T responses to MHV68 infection upon p50 deletion likely underlie the observed recrudescence in the lungs and increased viral load in the spleen at later times post-infection (Krug et al., 2009).

Non-canonical NF-κB signaling is highly active at multiple stages of B cell development, including entry into the germinal center (Batten et al., 2000; Kaisho et al., 2001; Senftleben et al., 2001; Claudio et al., 2002; Vora et al., 2003; Kalled, 2006; Mills et al., 2007). However, the role of the non-canonical pathway in MHV68 latency establishment is not well understood. Deletion of BAFFR, which signals through the non-canonical NF-κB signaling pathway, impaired B cell maturation and germinal center formation (Batten et al., 2000; Kalled, 2006). MHV68 infection of BAFFR^-/-^ mice led to reduced latency establishment and lower titers of virus-specific IgG (Frederico et al., 2014b).

Germline deletion of NF-κB components are associated with altered pathogenesis in the context of a spectrum of immunodeficiencies, demonstrating the importance of lymphocyte development and function in the establishment, and long-term control, of viral latency. However, such complex phenotypes complicate the determination of the cell-intrinsic roles of NF-κB signaling for MHV68 latency. Alternative experimental approaches been used to separate global immune defects from cell intrinsic effects on MHV68 pathogenesis. A recombinant MHV68 engineered to express a dominant negative form of the IκBα protein (MHV68-IκBαM) was used to block canonical NF-κB signaling only in the infected cell. This blockade led to a significant reduction of latency establishment in the lung and splenic B cells of infected mice (Krug et al., 2007). This defect could not be rescued by overexpression of the anti-apoptotic protein Bcl-2, suggesting that blockade of canonical NF-κB signaling led to a defect in latency rather than a defect in cell survival (Krug et al., 2007). The reduction of latency in the spleen was durable and accompanied by a reduction in virally infected B cells of the lungs (Krug et al., 2007). Specific inhibition of genes in the infected cell has also been accomplished using expression of Cre recombinase from the virus (Siegel et al., 2010) and transgenic mice, although this has not been used to probe the role of NF-κB components. The absence of global immune defects and the relative ease of recombinant virus generation makes this an attractive option to tease apart cell intrinsic roles for NF-κB as well as other signaling pathways.

Another method to examine cell-intrinsic roles during pathogenesis while avoiding global immune disruption is the generation of mixed bone marrow chimeric mice. WT stromal cells and a ∼50–80% proportion of WT bone marrow allow for the establishment of a normal immune system while setting the stage for a rigorous competition between a WT and mutant cell-type of interest for supporting viral latency. In a study of mixed bone marrow chimeras, MHV68 failed to establish latency efficiently in the NF-κB1 p50^-/-^ B cells as compared their WT B cell counterparts (Krug et al., 2009). In addition there was a more rapid decline in genome+ cells that lacked p50. The p50^-/-^ B cells suffered from an intrinsic defect in germinal center participation that correlated with the reduction of splenic latency. In addition to the establishment defect in the absence of p50, the persistence of pre-formed infectious virus in the lungs of the mixed bone marrow chimeras long after normal clearance of acute phase replication supports a role for NF-κB transcription factors in the maintenance of latency (Krug et al., 2009).

Abrogation of upstream signaling components also impairs latency. Infection of mixed MyD88^-/-^ and WT bone marrow chimeras revealed a defect in latency establishment in the MyD88^-/-^ B cells that was accompanied by a decrease in activation, germinal center participation, and Ig isotype class switching as compared to WT B cells (Gargano et al., 2008). Infection of mixed CD40^-/-^/WT chimeras revealed a defect in the long-term maintenance of latency in CD40^-/-^ but not WT cells (Kim et al., 2003). Although the frequency of cells harboring the virus was indistinguishable at 16 dpi, CD40^-/-^ B cells were present at reduced frequencies in the germinal centers and the number of CD40^-/-^ B cells harboring latent virus decreased at late times after infection (Kim et al., 2003). The deterioration in latency observed in the bone marrow chimeras implies that CD40 engagement and recruitment to the germinal center is important for homeostatic maintenance in the memory B cells population (Flano et al., 2003; Kim et al., 2003; Willer and Speck, 2005).

Extrinsic stimulation of latent cells with TLR agonists led to mixed reactivation outcomes. Stimulation of TLRs 3, 4, 5, and 9 induced reactivation from the A20-HE MHV68+ latent cell line (Gargano et al., 2009). In contrast, TLR7 and TLR9 stimulation of the latent S11 cells line led to a NF-κB p65-dependent suppression of reactivation (Haas et al., 2014). Administration of LPS (TLR4), R848 (TLR7), or CpG (TLR9) to infected mice led to heightened reactivation of latent splenocytes in the infected animal (Gargano et al., 2009), and CpG injection led to an increase in the frequency of splenic latency in pups and adult mice (Ptaschinski et al., 2010). The deletion of TLR3, TLR7, or TLR9 or the combination of TLR2 and TLR9 did not alter latency establishment (Gargano et al., 2008, 2009; Haas et al., 2014; Sun et al., 2015), but the TLR9^-/-^ mice were observed to have an increase in reactivation from latency (Haas et al., 2014). Interestingly, *ex vivo* treatment of splenocytes with BAFF slightly reduced reactivation of MHV68 from WT splenocytes (Williams et al., 2015). NF-κB signaling is likely involved in these phenotypes, but its direct role has not been validated.

### Synopsis and Future Mechanistic Investigations of NF-κB Signaling as a Host Determinant of Pathogenesis

Taken together, canonical NF-κB subunit activation is dispensable for MHV68 replication in fibroblasts and immortalized lung epithelial cells in culture, yet critical for control of virus replication in the lung and latency establishment in the spleen (Krug et al., 2007, 2009, 2010). Inhibition of NF-κB signaling via loss of membrane receptors or downstream signaling components impacts latency at different stages of chronic infection that coincide with B cell activation, germinal center differentiation, and the formation of long term memory B cells (Kim et al., 2003; Willer and Speck, 2005; Krug et al., 2007, 2009; Gargano et al., 2008). Albeit less investigated, non-canonical signaling also contributes to B cell functions that are conducive to MHV68 latency (Frederico et al., 2014b). Recent studies have revealed the complexity of latency establishment; there are numerous rounds of infection in multiple cell types and B cell subsets prior to the infection of, and transit through, the germinal center (Gaspar et al., 2011; Frederico et al., 2014a). Most of the studies that examine NF-κB signaling components have not defined the cellular origin of the block in latency establishment. Finally, engagement of TLR receptors influences reactivation from latency, albeit with variable outcomes (Gargano et al., 2009; Haas et al., 2014).

What remains unclear is the mechanism for these defects. Are these phenotypes due to a defect in B cell biology and/or a dysregulation of the viral latency program? We propose that gHVs have usurped the NF-κB signaling that transpires in B cells to promote the viral latency program. Thus, it becomes important to determine if specific viral genes are directly under the control of NF-κB subunits, and if specific NF-κB binding sites or responsive elements in the regulatory regions of viral genes mediate this regulation. This knowledge is critical to target those regions of the viral genome for mutagenesis and characterization *in vivo*. By this approach, the field can genetically separate the role of NF-κB in B cell biology from its function as a regulator of gHV gene regulation *in vivo*.

## Gammaherpesvirus Modulation of the NF-κB Signaling Pathway

### Modulation by EBV and KSHV

The gHVs encode numerous proteins that modulate or activate NF-κB signaling during both latent and lytic infection (Grossmann et al., 2006; Soni et al., 2007; Martin et al., 2008; Raab-Traub, 2012). During latency, EBV encodes two proteins, latent membrane protein 1 and 2 (LMP1/2), that mimic B cell signals that are involved in germinal center reactions and the process of memory B-cell differentiation. The EBV encoded LMP1 protein is a constitutively active CD40 receptor homolog and activates both canonical and non-canonical NF-κB pathways to promote the survival and proliferation of infected B cells (Uchida et al., 1999; Grimm et al., 2005; Le Clorennec et al., 2008; Ersing et al., 2013; Zhao et al., 2014). LMP2A has dual roles in promoting NF-κB signaling via Syk and Lyn kinases and enhances LMP1 signaling by controlling LMP1-interacting cellular TRAF2 transcription (Dawson et al., 2001; Guasparri et al., 2008). When expressed as single transgenes in mice, these molecules can promote B cell proliferation and antibody production, independently of germinal center formation (Rastelli et al., 2008; Minamitani et al., 2015). However, it is not clear what the impact of LMP1 and LMP2A activity are on B cell selection in the normal course of an EBV infection since the B cells of double LMP1/LMP2A transgenic mice were phenotypically normal (Hadinoto et al., 2008; Vrazo et al., 2012). EBV lytic genes may also impact NF-κB signaling. The viral dUTPase encoded by BLLF3 signals through TLR2 in a MyD88-dependant fashion (Ariza et al., 2009). In addition, the EBV protein BGLF4 encoded by ORF36 is a viral kinase that has been shown to phosphorylate cellular UTX to inhibit NF-κB signaling and promote RTA transactivation of lytic reporters (Chang et al., 2012).

Kaposi’s sarcoma-associated herpesvirus encodes multiple factors that activate NF-κB signaling, including the viral-FLICE inhibitor protein (vFLIP), viral G protein-coupled receptor (vGPCR), K1, K15, and ORF75. Cells latently infected with KSHV require activation of NF-κB signaling for survival and lymphomagenesis (Keller et al., 2000; Guasparri et al., 2004). KSHV vFLIP expression during latency drives both canonical and non-canonical NF-κB activation in order to promote cell survival and proliferation, as well as induce alterations in morphology (Chaudhary et al., 1999; Liu et al., 2002; Field et al., 2003; Matta and Chaudhary, 2004; Grossmann et al., 2006; Guasparri et al., 2006). The lytic KSHV vGPCR gene product is constitutively active and drives NF-κB signaling and the expression of proinflammatory chemokines and cytokines (Arvanitakis et al., 1997; Kirshner et al., 1999; Schwarz and Murphy, 2001). The role of K1 as an NF-κB activator is cell-specific; its expression promotes NF-κB signaling in B cells, yet inhibits it in HEK293 cells (Prakash et al., 2002; Konrad et al., 2009). K15 is a transmembrane protein that is expressed at low levels during latency and activates multiple pathways including NF-κB signaling (Brinkmann et al., 2003; Wang et al., 2007). Replacement of EBV LMP2A, which acts as a constitutively active BCR, with K1 and K15 rescues the ability of EBV to establish immortalized LCL lines (Steinbruck et al., 2015). KSHV miR-K1 targets the inhibitor IκBα to maintain NF-κB subunit activation and promote a latent state in PEL cells (Lei et al., 2010). The KSHV ORF75 tegument protein was identified in an *in vitro* screen for NF-κB activators (Konrad et al., 2009). MHV68 ORF75C and KSHV ORF75 are tegument proteins that have been recently found to activate RIG-I to promote NF-κB signaling (He et al., 2015), and are discussed further below.

### NF-κB Signaling Is Usurped by MHV68

During the herpesvirus lytic life cycle, viral gene expression follows an ordered cascade beginning with immediate early (IE), to early (E) and then late (L) genes. The IE protein, RTA is encoded by open reading frame 50 (ORF50) and is a conserved gHV transcription factor (Sun et al., 1998; Lukac et al., 1999; Feederle et al., 2000; Wu et al., 2000; Goodwin et al., 2001). RTA initiates lytic replication or the switch from latency to lytic replication by regulating transcription of E and L lytic genes. For the *Rhadinoviridae* γ2-herpesviruses, KSHV, RRV, HVS, and MHV68, RTA has been shown to be necessary and sufficient to initiate viral lytic replication during *de novo* infection (Sun et al., 1998; Feederle et al., 2000; Pavlova et al., 2003; Xu et al., 2005). Moreover, ectopic expression of RTA in quiescently infected cells disrupts latency and induces lytic reactivation (Lukac et al., 1998; Sun et al., 1998; Gradoville et al., 2000; Wu et al., 2000; Goodwin et al., 2001). The *Lymphocryptovirus* γ1-herpesvirus, EBV, requires the viral transactivator protein ZTA (BZLF1) for lytic replication, and RTA (BRLF1) serves as a critical co-factor (Ragoczy and Miller, 2001).

A number of cellular proteins such as IRF4 and HMGB-1 have been identified as co-factors that synergize with MHV68 RTA to regulate RTA-responsive promoters (Song et al., 2004; O’Flaherty et al., 2014). However, some cellular factors have been reported to inhibit gHV lytic replication by inhibiting RTA expression or activity, thereby promoting latency. For instance, cellular factors such as PARP-1, KSHV RTA-binding protein (K-RBP), and NF-κB can antagonize RTA to inhibit lytic replication (Brown et al., 2003b; Gwack et al., 2003; Yang and Wood, 2007). High levels of NF-κB p65 have a deleterious effect on gHV lytic replication, blocking lytic gene transcription and viral replication (Gutsch et al., 1994; Brown et al., 2003a).

**FIGURE 5:**
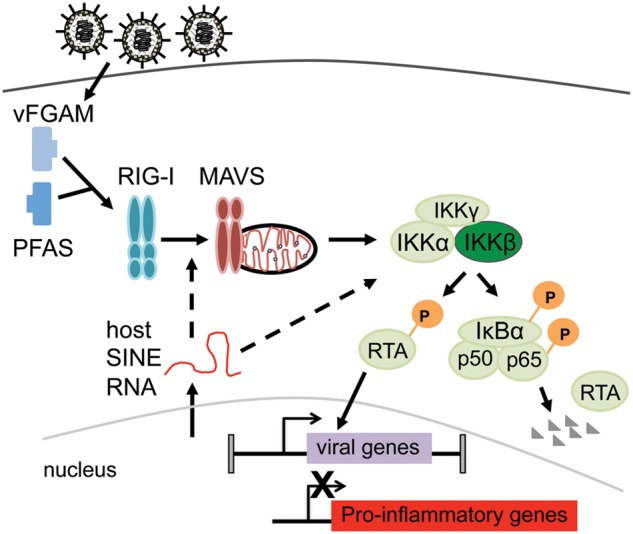
**Model for the role of canonical NF-κB signaling in productive infection.** Model summarizing recent reports of virus interplay with canonical NF-κB signaling to promote productive infection. Viral tegument protein (vFGAMS/vGAT) encoded by ORF75C and host SINE RNAs induced by infection leads to IKKβ activation and phosphorylation of RTA (Dong and Feng, 2011; He et al., 2015; Karijolich et al., 2015). Phosphorylation of RTA increases transactivation of viral genes (Dong and Feng, 2011). RTA targets NF-κB subunit p65 for degradation to dampen pro-inflammatory gene expression (Dong and Feng, 2011; Dong et al., 2012).

Interestingly, the gHV lytic transactivator proteins inhibit NF-κB signaling during viral replication. For EBV, the major lytic transactivator Zta (BZLF1) inhibits transactivation functions of NF-κB p65 (Morrison and Kenney, 2004). MHV68 and KSHV RTA proteins have been demonstrated to target p65 for degradation (Brown et al., 2003b; Yang et al., 2008; Dong et al., 2012). MHV68 RTA activated in response to MAVS/IKKB leads to ubiquitin-mediated degradation of p65 that is phosphorylated on Ser_468_ (Dong and Feng, 2011; Dong et al., 2012). MHV68 LANA induces the ubiquitination and degradation of p65, preventing NF-κB-dependent gene expression in 293 cells (Rodrigues et al., 2009). It is not clear if the major consequence of RTA degradation of p65 is to impair NF-κB to promote lytic gene expression or limit the transcription of NF-κB dependent proinflammatory cytokines (Dong et al., 2010, 2012; Dong and Feng, 2011) (**Figure 5**). Although NF-κB signaling is initially targeted for degradation by viral molecules, NF-κB subunit activation has been observed at late timepoints, from 12 to 24 hpi, during productive replication in fibroblasts (Krug et al., 2007, 2010); the impact of this late stage activation is not known.

Herpesvirus virions contain a tegument layer between the viral nucleocapsid and the viral envelope. All gHVs encode one to three tegument proteins with an approximate 140 amino acid stretch of homology to a cellular enzyme critical for *de novo* purine biosynthesis. This enzyme encoded by the *Pfas* gene, the formyl-glycineamide-phosphoribosyl-amidotransferase (FGARATs/FGAMs) catalyzes the fourth step in the *de novo* purine biosynthetic pathway (Zhao et al., 2013). The viral homologs herein termed vFGAMs (also referred to as vFGARATs or vGAT) have not preserved the known active sites of the host enzyme and, as such, are not predicted to retain enzymatic FGARAT activity (Gaspar et al., 2008). So far three functions have been described for vFGAMs: ND10 antagonism, facilitation of capsid trafficking in the cytoplasm, and NF-κB activation (Tsai et al., 2015). In a recent study by He et al. (2015), MHV68 vFGAM/vGAT (ORF75C) and KSHV ORF75 were found to recruit host FGARAT/PFAS to deamidate RIG-I. In turn, activated RIG-I complexed with MAVS and resulted in the activation of the upstream NF-κB signaling kinase, IKKβ.

Kaposi’s sarcoma-associated herpesvirus and MHV68 RTA migrate at higher molecular weight than predicted based on their amino acid content, from 64 to 90 kDa for MHV68 and 76 to 110 kDa for KSHV (Lukac et al., 1999; Wu et al., 2001). Phosphatase treatment decreases KSHV RTA mobility to ∼90 kDa suggesting that phosphorylation contributes in large to the change in RTA protein mobility (Lukac et al., 1999). Mass spectrometry analysis of KSHV RTA identified serine/threonine residues that were phosphorylated. Replacement of residues 634 and 636 from serine to alanine impairs KSHV RTA transactivation and reduces reactivation (Tsai et al., 2012). MHV68 RTA is phosphorylated *in vitro* by the IKKβ kinase at groups of serine/threonine residues S_550_T_552_S_556_ (STS) or T_561_T_562_S_564_ (TTS). Alanine replacement of the residues impairs RTA transactivation and growth of a TTS mutant virus (Dong et al., 2010). Taken together, RTA phosphorylation plays a role in regulating RTA-mediated viral gene expression which impacts gHV lytic replication and reactivation.

Murine gammaherpesvirus 68 RTA phosphorylation by IKKβ enhances RTA transactivation function and promotes RTA ubiquitin-mediated deagration of p65 (Dong et al., 2010, 2012; Dong and Feng, 2011) (**Figure 5**). Since IKKβ also phosphorylates the NF-κB subunit p65, RTA driven p65 degradation would presumably serve to limit NF-κB-dependent inflammatory cytokine production (Dong et al., 2010; Dong and Feng, 2011; He et al., 2015). An increase in pro-inflammatory cytokine production and lung inflammatory infiltrate was reported for MAVS^-/-^ mice during acute MHV68 infection (Dong and Feng, 2011; Dong et al., 2012). Interestingly, the kinetics of virus replication were also dramatically altered in the lungs in the absence of MAVS. Given that overexpression of p65 impairs MHV68 RTA transactivation (Brown et al., 2003b), the degradation of p65 might further enhance viral lytic gene expression as well.

Karijolich et al. (2015) recently identified another pathway that leads to IKKβ activation upon MHV68 infection. In this study, host short interspersed nuclear elements (SINE) RNAs were induced upon MHV68 infection. SINE RNAs drove IKKβ activation and subsequent phosphorylation of RTA to enhance its transactivation function. MAVS deficiency reduced the activation of IKKβ but did not abrogate it, suggesting that SINEs may activate NF-κB by both MAVS-dependent and independent means (Karijolich et al., 2015). Taken together, upon *de novo* infection of permissive cell lines, MHV68 activates upstream NF-κB signaling events to drive RTA phosphorylation to enhance lytic gene expression, but impairs downstream NF-κB subunit activation, likely to prevent inflammatory cytokine production (**Figure 5**).

### Evasion of Inflammasome

The inflammasome is an intracellular surveillance system that can respond to both damage-associated and pathogen-associated molecular patterns (DAMPs and PAMPs). By initiating these critical inflammatory responses, the inflammasome plays an important role in innate immune control of numerous pathogens, including double stranded DNA viruses (Rathinam et al., 2010; Sun et al., 2015). The inflammasome is both activated and subverted during human gHV infection in culture (Gregory et al., 2011; Kerur et al., 2011; Haneklaus et al., 2012; Ansari et al., 2013; Singh et al., 2013). Caspase-1 is the inflammasome effector protease that cleaves the proinflammatory cytokines IL-1β and IL-18. Surprisingly, mice deficient in *caspase-1* were not impaired for the control of acute replication of MHV68 in the lung or latency in the spleen (Cieniewicz et al., 2015). In contrast to other herpesviruses, infection with 10 infectious MHV68 particles per cell does not activate caspase-1 (Cieniewicz et al., 2015; Sun et al., 2015). The delivery of the ORF64 deubiquitinase as a tegument protein seemingly drives efficient shuttling of intact capsids to the nucleus, shielding the foreign viral genomic DNA from innate sensors that might trigger inflammasome activation upon infection (Sun et al., 2015). In addition, MHV68 infection caused a specific reduction in IL-1β production after extrinsic LPS stimulation or upon co-infection with *Salmonella enterica* Serovar Typhimurium (Cieniewicz et al., 2015). Interestingly, this impairment occurred at the proIL-1β transcript level without effecting *il18* and *tnfa* mRNA. IL-1β is regulated by NF-κB signaling, thus RTA was suspected to reduce IL-1β transcription via its function to degrade p65. However, the downregulation was independent of the lytic viral transcriptional activator RTA (Cieniewicz et al., 2015). Taken together, MHV68 impairs the inflammasome response by both inhibiting sensing and reducing IL-1β production during the initial stages of infection, likely via different mechanisms involving virion components and RTA-independent IE gene products.

## Impact of These Studies

Over the long course of coevolution with their hosts, the gHVs have evolved strategies to subvert host signaling and immunity, culminating in the establishment of a life-long infection that causes little deleterious effect to the host. However, in the context of immunosuppression, these latent infections predispose their host to the development of malignancies. Murine gHV infection of mice serves as a powerful system to investigate the complex interactions of virus and host factors that contribute to pathogenesis in a natural host. Impairment of NF-κB signaling through the use of knockout mice and recombinant viruses has revealed a critical role for this host pathway in the establishment of latency in the host B cell latency reservoir (**Table 2**). Targeted ablation strategies and experimental determination of the NF-κB-dependent gene expression profile of the virus and host are critical to identify roles intrinsic to the infected cell. With regard to productive replication, *de novo* infection with MHV68 leads to activation of upstream NF-κB signaling molecules to enhance transactivation of lytic gene expression and promote productive replication (**Figure 5**). A better understanding of the mechanism of action for the host factors of the infected cell and the molecular interface between the virus and host will identify novel therapeutic approaches to disrupt replication and latency, and ultimately enable the prevention or elimination of virus-driven malignancies.

## Author Contributions

All authors contributed to the writing and figure generation for the work.

## Conflict of Interest Statement

The authors declare that the research was conducted in the absence of any commercial or financial relationships that could be construed as a potential conflict of interest.
